# How Do Personality Dysfunction and Maladaptive Personality Traits Predict Time to Premature Discontinuation of Pharmacological Treatment of ADHD?

**DOI:** 10.1177/10870547241309524

**Published:** 2025-01-23

**Authors:** Peter Jacobsson, Tove Granqvist, Christopher J. Hopwood, Robert F. Krueger, Bo Söderpalm, Thomas Nilsson

**Affiliations:** 1 University of Gothenburg, Sweden; 2 Region Halland, Varberg, Sweden; 3 Trana Psykologer, Varberg, Sweden; 4 University of Zurich, Switzerland; 5 University of Minnesota, Minneapolis, USA; 6 Sahlgrenska University Hospital, Gothenburg, Sweden

**Keywords:** adult ADHD, alternative model of personality disorder, pharmacological treatment, premature discontinuation, adherence

## Abstract

**Objectives:**

Non-adherence to medication is common in the adult ADHD clinical group. The goal of this pre-registered study was to examine whether the DSM-5 Alternative Model of Personality Disorder (AMPD), generality personality dysfunction (LPFS-BF 2.0) or maladaptive personality traits (PID-5), can predict time to premature discontinuation of pharmacological treatment beyond other known factors.

**Methods:**

A sample of 284 adult patients with ADHD (60.6% female; *M*_age_ = 32.31 years) were investigated for medication adherence from 2018 to 2023, using time-to-event analytic methods.

**Results:**

Of the sample, 54 were found to have discontinued treatment prematurely without consulting their physician. Interestingly this group was prescribed considerably lower doses before discontinuation than adhering patients. General personality dysfunction and maladaptive antagonistic personality traits are implicated in varying degrees, with the specific maladaptive personality facets *Intimacy Avoidance* and *Deceitfulness* (PID-5) significantly predicting time to premature discontinuation of ADHD medication beyond other known reasons for non-adherence.

**Conclusions:**

The broader implication is that the emerging personality pathology models hold promise to predict non-adherence in the adult ADHD population.

## Introduction

ADHD is a common disorder which affects 2% to 5% of the adult population (Kessler et al., 2006; Simon et al., 2009; Song et al., 2021). Key dysfunctions in adult ADHD include difficulties in organization, particularly attending to mundane, yet important, life-activities (Barkley & Murphy, 2011; Weiss, 2022). Perhaps not surprisingly, discontinuation of pharmacological treatment and having difficulties in adhering to treatment protocol, is common in the ADHD population, with studies showing that 13,2 - 64% of adult ADHD patients have typically dropped out after 230 days (Brancati et al., 2023; Gajria et al., 2014; Schein et al., 2021). However, a recent large observational study by Brikell et al (2024) indicated that variability for time to discontinuation is high depending on age and country studied, with Sweden at the higher end with median first discontinuation at 411 days and with 39% to 48% of adults remaining on medication after a year. Attrition continues, however, with only 11% to 15% still on medication after 5 years (Brikell et al., 2024). There is also evidence from a large meta-analytic study that adults show reduced tolerance to ADHD medication and derive less effect from the treatment than children and adolescents (Cortese et al., 2018).

Available pharmacological treatment for ADHD however, is efficacious (Cortese et al., 2018). Thus identifying causes for discontinuation and non-adherence to medication is important so that appropriate interventions can be developed if continued medication is warranted (Parkin et al., 2022). ADHD symptomology and its inherent organizational difficulties by itself does not fully explain the variability in medication adherence among afflicted individuals (Parkin et al., 2022). This underlines the importance of overcoming the challenges of finding optimal types of medication, dosages, and formulations early in the treatment process.

The most common reasons for discontinuation are adverse medication effects, or not sufficient effect, but other identified relationships for non-adherence include gender, younger age, medication history, and comorbid psychiatric conditions (Mohr-Jensen et al., 2020). Different samples, substances, and methodologies have made it difficult to compare results across studies (Brikell et al., 2024).

Relevant to the current study, comorbid psychiatric conditions have also been shown to predict poorer outcomes (Lensing et al., 2013), and specifically comorbid Antisocial Personality Disorder, or more than one personality disorder, had a significant impact on treatment adherence (Robison et al., 2010; Torgersen et al., 2012). However, the ten possible personality disorders except for Borderline Personality Disorder, are still currently underdiagnosed in psychiatric settings, thus making categorical personality disorder diagnosis as an indicator, currently not sufficiently clinically useful (Beckwith et al., 2014).

The dimensional emerging models of personality disorder in the DSM-5 and the ICD-11 may present more viable alternatives to identify indicators related to non-adherence (American Psychiatric Association [APA], 2013; World Health Organization [WHO], 2019 [2022]). The models consist of a primary assessment of general personality dysfunction that is graded from: no dysfunction to severe dysfunction, as well as an individual descriptive profile of five maladaptive personality traits (Bach & First, 2018; Hopwood et al., 2011; Krueger & Markon, 2014). These models are being implemented exponentially in psychiatric clinics worldwide (Bach et al., 2022). General personality dysfunction has been proposed to be the key element for assessing personality disorder in the recently published ICD-11 (ICD-11 PD; WHO, 2019 [2022]), with trait assessment as an optional element.

Personality pathology is an important factor in predicting psychological difficulties in many areas of life, such as personal and work-related relationships, achieving important life goals, somatic health, and risk exposure (Dixon-Gordon et al., 2015; Hengartner et al., 2014; Hopwood et al., 2011; Sansone & Sansone, 2010). Although not sufficiently investigated, personality pathology is likely to be distributed throughout the ADHD population, and individuals with higher degrees of personality pathology are also more impaired in other areas of life-functioning (Jacobsson et al., 2021). In the current study, the constructs and measures emanate from the DSM-5 Alternative Model of Personality Disorders (AMPD) in which criterion A, the general *severity* of dysfunction is operationalized in the Levels of Personality Functioning Scale (LPFS; Bender et al., 2011; Morey et al., 2011). The LPFS includes the four elements of *Identity*, *Self-direction*, *Empathy*, and *Intimacy* to be evaluated separately, and then to generate a unitary score on a five-level scale between no dysfunction to extreme dysfunction. There are currently several measures to assess LPFS, both as self-reports and structured interviews (Hopwood et al., 2018; Hummelen et al., 2021; Hutsebaut, Feenstra et al., 2016; Hutsebaut, Kamphuis et al., 2016; Waugh et al., 2021). The Levels of Personality Functioning Scale- Brief Form (LPFS-BF 2.0) was the first published self-report instrument designed to measure the LPFS and has been shown to have robust psychometric properties and clinical utility, as well as high correlations with other more extensive instruments (Hutsebaut, Feenstra et al., 2016; Waugh et al., 2021). Because of its clinical utility and strong psychometric properties, the LPFS-BF 2.0 is recommended in the International Consortium for Health Outcomes Measurement (ICHOM) standard set for outcome measures for personality disorders (Prevolnik Rupel et al., 2021).

To assess maladaptive personality traits according to criterion B in the AMPD, the Personality Inventory for DSM-5 (PID-5) is commonly used, organizing the responses into the five higher order maladaptive personality domains: *Negative Affectivity*, *Detachment*, *Antagonism*, *Disinhibition*, and *Psychoticism*. To increase specificity when needed, 25 lower order facets such as *distractibility*, *impulsivity*, and *irresponsibility* are nested within the Disinhibition domain. The other four higher order maladaptive domains can similarly be divided into three, or more, lower order personality traits for higher resolution. The five domains are related to the established Five-Factor Model (FFM; Costa & McCrae, 1995), where Negative Affectivity is consistent with Neuroticism; Detachment with low Extraversion; Antagonism with low Agreeableness; Disinhibition with low Conscientiousness; and Psychoticism weakly related to Openness of Experience (Zimmermann et al., 2019).

Specifically, the construct of general personality dysfunction, operationalized with LPFS in the Alternative Model of Personality Disorder, has been shown to have a large impact on functioning in important life domains (Buer Christensen et al., 2020; Ohse et al., 2023). Although the notion of dimensional personality pathology severity is not new, the absence of the construct in official diagnostic models has also been reflected in the dearth of previous research on how it affects medication adherence. However, using the number of diagnosed personality disorders as a proxy for severity, or psychodynamic constructs of personality functioning, there seems to be some indications for medication non-adherence (Gift et al., 2016; Haller et al., 2022).

Even though we have not found any studies linking the maladaptive personality traits included in the DSM-5 Alternative Model of Personality Disorder or the dimensional ICD-11 Personality Disorder model, there is a somewhat more substantial body of research linking normal range personality traits to medication adherence in a wide variety of underlying conditions: The Big Five personality trait *Conscientiousness* is consistently strongly correlated to medication adherence, either with individuals high in Conscientiousness related to increased adherence, or individuals low in Conscientiousness predicting non-adherence (Axelsson et al., 2011; Emilsson et al., 2011; Molloy et al., 2014). Since the core difficulties defining the ADHD population are directly related to low Conscientiousness (or high *Disinhibition*) and thus already expecting problems with medication non-adherence, personality predictors are expected to present differently (Jacobsson et al., 2021). For example, Emilsson et al (2020) found that low *Agreeableness* (high *Antagonism*) was correlated to non-adherence behavior among Swedish adolescents.

There is currently a general scarcity of research on ADHD and the dimensional models of personality disorder; and as far as we are aware, there are no studies on how these relate to adherence to ADHD medication (Jacobsson et al., 2021; Smith & Samuel, 2017). It would seem reasonable from the discussion above, that particularly general personality dysfunction and antagonistic personality traits could impact medication adherence, independent of ADHD symptomatology.

We therefore aim to explore the relationships between degree of general personality dysfunction, maladaptive traits, and premature discontinuation (PMD) of ADHD medication in the adult ADHD population. Previously known variables that may impact medication adherence, such as those stated above, are also included in our analyses. Significant relationships will provide an important and convenient way of tailoring individualized interventions to increase pharmacological effectiveness. Specifically, we aim to investigate whether: (a) general personality dysfunction, measured with Levels of Personality Functioning Scale- Brief Form (LPFS-BF 2.0), and (b) specific maladaptive personality expressions, measured by Personality Inventory for DSM-5 (PID-5), predict premature termination of pharmacological treatment in an adult population diagnosed with ADHD.

## Method

### Open Practices

The study and the analysis plan were formally pre-registered in: https://osf.io/tsz2h/?view_only=9a4ba3f090644634803e112dcf2055f6

### Setting and Recruitment

There are six adult outpatient public health care psychiatric clinics in Halland that provide care and treatment for individuals suffering from more severe forms of psychiatric disorders, including a growing number of neurodevelopmental disorders, such as adult ADHD. Adult individuals with an ADHD diagnosis currently form the largest diagnostic category at 49% in 2023; compared to personality disorders only at 4%. This situation is not uncommon in an international perspective (Choi et al., 2022; Mulder, 2021). The individuals selected in this study have undergone an ADHD assessment after referral. The patients were referred either by other health care professionals, or by self-referrals and subsequent psychiatric triage. The assessment is according to the regional guidelines and has a standard protocol of; (a) history taking, both from the patient and if possible, also from an informant; (b) includes a minimum battery of self-reports on ADHD symptoms, life-function, and general psychiatric symptomatology, general personality dysfunction, and maladaptive personality traits. From 2018 to 2023 an online intake system has been implemented in which both the LPFS-BF and the PID-5 are part of the digital ADHD assessment package administered to patients. In some cases, the PID-5 has been omitted by individual assessors due to the administrative burden for specific individuals. Due to effectiveness requirements, additional assessments are included only if deemed necessary. These commonly include structured interviews of ADHD symptoms and impairment, as well as in-depth interviews of the severity of personality pathology, and in several cases also neuropsychological evaluations (Conners et al., 2018; Ramos-Quiroga et al., 2019; Sheehan et al., 1998; Wechsler, 2008). Although clinical psychologists are mainly responsible for the assessment process, all diagnoses are confirmed by a psychiatrist before treatment initiation. Diagnosis is reached if criteria according to DSM-5 are fulfilled: (a) thresholds of symptoms are met (currently five of nine possible symptoms either of inattention and/or hyperactivity/impulsivity); (b) several symptoms were present before 12 years of age; (c) symptoms are present and associated with impairment in two or more important life domains; (d) not better explained by other somatic or psychiatric disorders (APA, 2013).

Comorbidity is ubiquitous in ADHD in the current classification paradigm, so a detailed rationale is required in the assessment guidelines to describe whether other psychological issues are seen as co-occurring or are the basis for another diagnosis instead of ADHD. Finally, the guidelines also require a reflective evaluation of the reliability of the diagnostic procedure in which the evaluator states if the diagnosis has moderate to very high reliability. Anything lower than moderate does not convert into a diagnosis. Since this study has a naturalistic design, in which the evaluating psychologists have followed standard assessment protocols that provide flexibility concerning assessment instruments, not all assessed individuals have been included in the material, or even approached for consent. Possible selection bias is thus limited to individual psychologists and specific assessments in which instruments relevant to this study have not been deemed necessary or possible. However, the relevant personality self-reports, the LPFS-BF, and the PID-5 were administered prior to consent. More systematic bias is also due to individuals who, because of intellectual, motivational, or language difficulties, have not succeeded in filling out the relevant self-reports. Psychologists asked patients who were referred for ADHD evaluations and had undergone the standard assessment procedure for consent to participate. All participants were informed of the study and signed an informed consent.

Clinical patient documentation in existing progress notes have been explored manually to extract variables. All included subjects have initiated medication. Extraction was performed by a master’s level psychologist (TG) accomplishing her specialization program, who was blind to separate data files with personality data. Administering psychiatrists and psychiatric nurses were blind to personality data.

Variables extracted from patient documentation according to the pre-registration protocol were: (a) Date when medication was initiated; (b) Date(s) when medication was discontinued and if documented the reason for discontinuation; (c) Date when contact with the clinic was discontinued and, if possible, the reason for discontinuation; (d) Medication type and dosage; (e) Comorbid psychiatric diagnosis including SUD; (f) Side-effects when documented. Premature discontinuation (PMD) was recorded (a) when the individual informed the administering psychiatrist or the psychiatric nurse that they wished to discontinue the ADHD medication; (b) when there was indicated from patient records that discontinuation had occurred from lack of renewing medication or being absent from follow-up visits at the unit with psychiatrists or psychiatric nurses. Subjects were not recorded as PMD if they had moved, left the clinic or become pregnant. None of the individuals in the PMD group resumed their medication within 12 months.

We standardized the stimulants into a daily dose of amphetamine equivalents in mg (amphetamine equivalents: equal approximately to the methylphenidate dose (mg) divided by 2 and the Elvanse^®^ dose (mg) divided by 3.3) to enable clustering of different medication types and excluded non-stimulants. This sub-sample included 207 individuals (PMD: 42; Continued: 165) who had completed both personality measures and were medicated with some form of stimulant.

The sampling started in 2018 and the cutoff date was September 12, 2023. All participants were adults from 18 years, initially drug naïve to stimulant medication, and assessed as fulfilling ADHD criteria based on the assessment.

Ethical approval has been granted by the Ethics Review Board in Lund, Sweden, Dnr: 2018/53 for the overarching dissertation project of which this study is a part of; and the study is conducted in accordance with the current Declaration of Helsinki.

### Measures

The *Level of personality functioning scale, brief form* (LPFS-BF 2.0; Hutsebaut, Feenstra et al., 2016; Weekers et al., 2019) measures personality functioning. Also briefly described above, the underlying concept of general personality dysfunction operationalized by Levels of Personality Functioning Scale (LPFS) indicates clinically significant commonalities across all personality disorders and consists of impairments in self- and interpersonal functioning (Bender et al., 2011; Morey et al., 2011). The LPFS-BF 2.0 has 12 items rated on a 4-point Likert scale from 1 (completely untrue) to 4 (completely true), resulting in a global score reflecting the general severity of personality pathology. The LPFS-BF has shown acceptable psychometric properties (Hutsebaut, Feenstra et al., 2016; Prevolnik Rupel et al., 2021; Weekers et al., 2019, 2022). Internal consistency for the LPFS-BF 2.0 across our sample was α = .85, which is consistent with other studies (Hutsebaut, Feenstra et al., 2016; Weekers et al., 2019; see Supplemental Table C).

*The Personality Inventory for DSM-5 (PID-5*; APA, 2013; Krueger et al., 2012) measures maladaptive personality traits. Internal consistency of the PID-5 is acceptable to high depending on length of domain relative to facet scales (Zimmermann et al., 2014, 2019). The facets appear, for the most part, to be unidimensional. Higher interstitiality of some facets led to the reduction of the number of facets included in each domain, so that the APA recommended that only 15 of the 25 facets comprise the basis for the 5 higher order domains (APA, 2013). We used the APA recommended domain scoring procedure involving three facets per domain. Cronbach’s alphas for domains in our sample spanned from α = .83 (Disinhibition) to α = .94 (Psychoticism), and for facets: α = .56 (Irresponsibility) to α = .94 (Depressivity; see Supplemental Table C).

### Analyses

Data analyses were conducted in IBM SPSS Statistics (Version 29).

Following our plan according to the pre-registration, first we computed relevant descriptive statistics of our sample (premature discontinuation vs. fully continued at study end point). Statistical analysis of group differences on demographic variables, skewness and kurtosis values of LPFS-BF 2.0 and PID-5 were investigated by calculating Cohen’s *d* as an estimate of effect size, and 95% confidence intervals around these estimates. Statistical significance was estimated at .05. Due to the relatively small samples and the large number of non-hypothesized tests, we focused primarily on effect sizes rather than *p*-values.

The second step in our preregistered analytic plan was to use Cox Hazard Ratio analyses with ‘event’ defined as premature termination of medication over the time period 2018 with first included subject until last registered included subject September 2023. Survival time unit is number of days until termination or right censoring, coded 0 = censoring or 1 = terminal event observed (i.e., discontinuation). For clarity reasons the censored group is called *Continued* and the group that terminated is called *Prematurely discontinued (PMD)*. Separate analyses were performed with; (a) general personality dysfunction (total sum LPFS-BF 2.0), (b) maladaptive personality domains: (1) Negative Affectivity, (2) Detachment, (3) Antagonism, (4) Disinhibition, and (5) Psychoticism (PID-5 domain scores) as well as the 25 lower order personality facets (PID-5 facet scores) treated as independent variables.

Post hoc sensitivity analyses of; (a) medication effects, (b) psychiatric comorbidity, (c) side-effects, (d) sex, and (e) age. Medication effects were assessed using a heuristic conversion from the types of stimulants used to a daily dose of amphetamine equivalents. Non-stimulants such as atomoxetine and guanfacine thus were excluded in these analyses. The recommended maximal dosages of stimulants were identified using the Swedish medicines information portal (Fass.se). Dosages between the two groups were analyzed both as *continuous* variables (mean dose levels) and as *binary* constructs (proportion reaching max doses). Psychiatric comorbidity was measured both as a *binary* construct, whether any other psychiatric diagnoses, other than ADHD, were present at the point of assessment, and also how *many* diagnoses were documented for each individual. Side-effects were assessed as present or not present, based on documented reports from patients in the progress reports. These covariates were first analyzed identically to the personality variables and then added to Cox regression multivariate analyses to explore the possible impact for implicated personality variables (see Supplemental Table D).

## Results

A total of 284 patients had been included of which 54 had discontinued prematurely and not resumed medication for at least 180 days. A total of 407 individuals were assessed and initiated pharmacological treatment during the sampling period. Thus, 70% of the assessed and treated sample consented to participate in the study. Of the total included sample six individuals became pregnant during medication and thus paused medication with intention to resume and were placed in the continued group. Seven individuals moved from the region and the monitoring clinics and were also coded as continued in the material.

The total sample consists of 284 included individuals, but due to the naturalistic nature of the study, not all included had completed both of the personality questionnaires LPFS-BF 2.0 and the PID-5. For the LPFS-BF 2.0, 267 individuals with ADHD had completed the form and 231 had completed the PID-5. A total of 216 individuals (PMD: 46; Continued: 170) had completed both personality inventories. In the total sample 60.6% were female, and the mean age was 32.3 (*SD* = 10.1, range 18–61 years).

The most common co-occurring psychiatric diagnoses in the sample were Anxiety Disorders (37.2%) and Depressive Disorders (27.4%). Less frequent diagnoses were PTSD and Autism Spectrum Disorder, both at 6%; Personality Disorders (5.6%), Substance Use Disorders (3.5%), OCD, and Eating Disorders, both at 3.2% (see Supplemental Table A).

In this study, 21% discontinued their ADHD medication without previous agreement with their psychiatrists. Most individuals were medicating with stimulants and only 5% with the non-stimulants atomoxetine or guanfacine. Mean time before discontinuation was 468 days. The PMD group discontinued earlier with dosages much more modest than in the continued group. The mean time in the continued group was 708 days, compared to 468 days in the PMD group (Cohen’s *d* = .71; *p* < .001).

**Figure 1.**
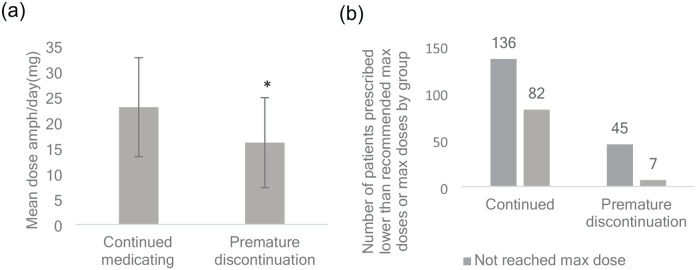
(a) Mean stimulant daily dose expressed in amphetamine equivalents (mg ± *SD*) in the continuously medicating group and in the prematurely discontinuing group. *p < .001 versus continuous medication. (b) Number of patients prescribed or not prescribed the recommended max dose of stimulant (or higher). A significantly lower proportion was prescribed the recommended max dose in the prematurely discontinuing group. Total *N* = , χ^2^ < .001. *Note.* Swedish medicines information portal (Fass.se) was used to identify max dosages for each stimulant.

There were no significant differences in age or gender between the group that discontinued prematurely and the group that continued (see Supplemental Table B). However, there were differences between the two groups concerning co-occurring psychiatric diagnoses (χ^2^ = 4.46; *p* < .05), side-effects (χ^2^ = 4.32; *p* < .05) and concerning the mean stimulant dose prescribed (Continued group 22.69 ± 9.74 mg vs. PMD 15.90 ± 8.80 mg, *d* = 0.71, *p* < .001, Figure 1a), as well as whether individuals were prescribed maximal recommended stimulant dosages (or higher), according to the Swedish pharmacopoeia (Fass.se; χ^2^ = 11.08; *p* < .001; Figure 1b). Thus, more co-occurring psychiatric diagnoses, more side-effects, and smaller dosages characterized the prematurely discontinued group.

Due to the stringent limits on prescribing stimulants, substance misuse may sometimes be a reason for enforced discontinuation. Ten individuals in our sample were diagnosed with a substance use disorder (SUD; four who discontinued and six who were continued at end of data collection), but there was no indication of premature discontinuation due to SUD (χ^2^ = 2.42; *p* = .12).

Differences between people who discontinued prematurely (54 individuals) versus continuing (230 individuals) ranged from 0 to 0.41 standard deviations across the LPFS-BF 2.0 global score; 25 PID-5 trait facets and 5 trait domains, with 10 values being larger than *d* = 0.20. Personality severity as measured by the LPFS.BF 2.0 had an effect size of 0.35; whereas PID-5 Antagonism was the only personality trait domain with an effect size >0.20 (0.36). However, only four group differences were statistically significant, the Antagonism domain and the facets *Deceitfulness* (*d* = 0.41), *Intimacy Avoidance* (*d* = 0.34), and *Hostility* (*d* = 0.39; Table 1).

**Table 1.** Differences Between People Who Continued Versus Prematurely Discontinued.

Personality dysfunction and maladaptive personality traits	*d*	CI 95%	*p*
**Personality dysfunction** (LPFS-BF)	**0.27**	[−0.03, 0.57]	.08
***Trait domains and facets*** (PID-5)			
**Negative affectivity**	0.00	[−0.32, 0.32]	1.00
* Emotional lability*	0.07	[−0.25, 0.39]	.66
* Anxiousness*	0.02	[−0.30, 0.33]	.92
* Separation insecurity*	−0.08	[−0.39, 0.24]	.62
Restricted affectivity	0.03	[−0.28, 0.35]	.84
Hostility	**0.39**	[0.07, 0.71]	.02
Perseveration	−0.07	[−0.39, 0.24]	.66
Submissiveness	−0.15	[−0.47, 0.16]	.34
**Detachment**	0.15	[−0.16, 0.47]	.35
* Withdrawal*	0.13	[−0.19, 0.45]	.42
* Anhedonia*	−0.10	[−0.42, 0.22]	.53
* Intimacy avoidance*	**0.34**	[0.02, 0.66]	.04
Depressivity	0.07	[−0.25, 0.38]	.67
Suspiciousness	0.08	[−0.23, 0.40]	.60
**Antagonism**	**0.36**	[0.04, 0.67]	.03
* Manipulativeness*	***0.24***	[−0.08, 0.56]	.14
* Deceitfulness*	**0.41**	[0.09, 0.73]	.01
* Grandiosity*	***0.27***	[−0.05, 0.58]	.10
Attention seeking	0.09	[−0.23, 0.40]	.59
Callousness	***0.27***	[−0.05, 0.58]	.10
**Disinhibition**	0.11	[−0.21, 0.43]	.49
* Irresponsibility*	***0.21***	[−0.11, 0.53]	.19
* Impulsivity*	0.19	[−0.12, 0.51	.23
* Distractability*	−0.17	[−0.49, 0.14]	.29
Rigid perfectionism (lack of)	0.03	[−0.28, 0.35]	.84
Risk taking	0.07	[−0.24, 0.39]	.66
**Psychoticism**	0.18	[−0.14, 0.49]	.27
* Unusual beliefs*	0.17	[−0.15, 0.48]	.31
* Eccentricity*	0.11	[−0.21, 0.42]	.51
* Perceptual dysregulation*	***0.23***	[−0.08, 0.55]	.15

*Note.* **Bold** signify correlations significantly different from zero (*p* < .05). **Bold** and *italicized* signify effect sizes above *d* = 0.2. *Italicized* facets are the primary facets included in the APA algorithms för higher order domains. Total *N* = 284; Personality Dysfunction *N* = 267 (prematurely discontinued *N* = 54; continued *N* = 213); Maladaptive Traits *N* = 231 (prematurely discontinued *N* = 49; continued *N* = 182). LPFS-BF = Levels of Personality Functioning Scale, Brief Form; PID-5 = Personality Inventory for the DSM-5.

Our primary analytic approach was time-to-event analyses, which produces hazard ratios for premature discontinuation in a survival framework. The hazard ratio can been defined as the ratio of risk of outcome in one group divided by the risk of outcome in another group, occurring at a given interval of time (Brody, 2016). Several variables had hazard ratios indicating 40% greater risk for premature discontinuation (Table 2), including Personality Dysfunction, Hostility, Intimacy Avoidance, and the Antagonism domain and its facets Deceitfulness, Grandiosity, and Callousness. However, only Intimacy Avoidance and Deceitfulness were statistically significant.

**Table 2.** Hazard Ratios of Premature Discontinuation.

Personality dysfunction and maladaptive personality traits	HR	CI 95%	*p*
**Personality Dysfunction** (LPFS-BF)	***1.44***	[0.94, 2.20]	.09
***Trait Domains and Facets*** (PID-5)			
**Negative Affectivity**	0.97	[0.61, 1.53]	.90
* Emotional Lability*	1.09	[0.75, 1.59]	.64
* Anxiousness*	0.97	[0.66, 1.41]	.87
* Separation Insecurity*	0.90	[0.63, 1.30]	.59
Restricted Affectivity	0.97	[0.63, 1.51]	.91
Hostility	***1.47***	[0.95, 2.28]	.08
Perseveration	0.82	[0.52, 1.30]	.40
Submissiveness	0.92	[0.61, 1.37]	.67
**Detachment**	***1.46***	[0.85, 2.52]	.18
* Withdrawal*	1.19	[0.81, 1.74]	.38
* Anhedonia*	0.91	[0.58, 1.42]	.67
* Intimacy Avoidance*	**1.88**	**[1.23**, **2.89]**	.**00**
Depressivity	1.11	[0.77, 1.60]	.58
Suspiciousness	1.14	[0.70, 1.86]	.61
**Antagonism**	***1.57***	[0.98, 2.50]	.06
* Manipulativeness*	1.23	[0.85, 1.78]	.28
* Deceitfulness*	**1.61**	**[1.05**, **2.45]**	.**03**
* Grandiosity*	***1.47***	[0.93, 2.32]	.10
Attention Seeking	1.04	[0.74, 1.47]	.81
Callousness	***1.41***	[0.86, 2.33]	.18
**Disinhibition**	1.07	[0.54, 2.11]	.84
* Irresponsibility*	1.27	[0.75, 2.16]	.37
* Impulsivity*	1.17	[0.74, 1.85]	.51
* Distractability*	0.71	[0.42, 1.21]	.21
Rigid Perfectionism (lack of)	0.93	[0.64, 1.34]	.68
Risk Taking	0.97	[0.57, 1.63]	.89
**Psychoticism**	1.17	[0.70, 1.95]	.55
* Unusual Beliefs*	1.21	[0.76, 1.95]	.42
* Eccentricity*	1.00	[0.70, 1.43]	.99
* Perceptual Dysregulation*	1.33	[0.80, 2.22]	.27

*Note.* **Bold** signify hazard ratios significantly different from zero (*p* < .05). **Bold** and *italized* signify hazard ratios over 40%. *Italicized* facets are the primary facets included in the APA algorithms for higher order domains. Total *N* = 284; LPFS-BF *N* = 267 (prematurely discontinued *N* = 54; continued *N* = 213); PID-5 *N* = 231 (prematurely discontinued *N* = 49; continued *N* = 182. LPFS-BF = Levels of Personality Functioning Scale, Brief Form; PID-5 = Personality Inventory for the DSM-5.

Sensitivity analyses with the more constricted samples described above that included only fully completed inventories, as well as with only individuals medicated with stimulants, confirmed these patterns (see Supplemental Tables E and F). Even when side-effects, amphetamine dosages, and co-morbidity were added to a multivariate Cox HR analysis, Intimacy Avoidance remained statistically significant. Moreover, even with the significant impact of not reaching max dosage in the PMD group, Intimacy Avoidance and Deceitfulness still remained statistically significant, *p* = .023; β = 1.68, 95% CI [1.08, 2.62] and *p* = .033, β = 1.64, 95% CI [1.04, 2.60], respectively.

## Discussion

In this study, we examined whether personality variables from the DSM-5 Alternative Model of Personality Disorder are related to medication compliance in adult patients diagnosed with ADHD. Our primary findings were that the personality traits *Deceitfulness* and *Intimacy Avoidance* predicted premature medication discontinuation. These results indicate that personality assessment can provide clinically important information in ADHD assessments. Indeed, the risk of premature discontinuation was nearly twice as high for individuals with elevated levels of these traits, and they persisted with factors such as psychiatric comorbidity, side-effects, and dosage controlled.

Our results are consistent with a recent paper by Emilsson et al. (2020) in which the authors investigated the complex relationships between adherence, personality traits, beliefs about medication, and perceptions of ADHD. The authors found that Antagonism was associated with intentional non-adherence. Our study both strengthens that finding and specifies that specific antagonistic features involving dishonesty may be predictive of discontinuing medication before reaching the desired medication effect (see below). In contrast, the effect of *Intimacy Avoidance* was not anticipated.

Both of these traits feature potentially maladaptive interpersonal beliefs and behavior involving mistrust and need for autonomy (Hopwood et al., 2013). The patient’s belief in the care provided by their physician is thought to be key for successful health care, and it is possible that individuals who find this difficult are less likely to follow medical advice (Hall et al., 2001; Khan & Aslani, 2021). Another possibility is that individuals who are deceitful may have also provided less accurate diagnostic data in the first place, and discontinued when they discovered the medication was not helpful. In contrast, people with intimacy avoidance problems may have found it difficult to attend follow-up sessions, and thus were willing to sacrifice medication to avoid them. Given the relatively small sample size, generalizability issues, and the fact that we did not make hypotheses about trait facets, these findings should be replicated and specific hypotheses about their mechanisms should be followed up in future research.

Also, a somewhat unanticipated and clinically interesting finding was the large discrepancy in dosages between the individuals that prematurely discontinued treatment and the group that adhered to medication, both in the continuous measures of dose as well as in the binary construct of reaching recommended max dosage or not. One explanation for this finding could simply be that these individuals for some reason do not require higher doses for effect, but an objection to this interpretation is the fact that they discontinued treatment. A more likely explanation would be that if individuals do not reach target doses in ADHD medication; they will not experience the intended effects and thus likely lose motivation to continue medication. These results are consistent with the linear relationship between medication adherence and dosages that was found in a study by Skoglund et al. (2016), albeit in a population of patients co-morbid with substance use disorder. Possibly the lower mean dose reached in the prematurely discontinued group was due to a slower titrating rate than in the adhering group or discontinuers did not adhere long enough to allow full titration. Although titration rate was not specifically explored in the present study, both these explanations can probably be ruled out, since the mean time to discontinuation (468 days) clearly was long enough to allow titration to optimal dosage. In clinical practice, this titration is usually done within 6 months. Yet another explanation for the lower doses in the prematurely discontinued group could be that they were more sensitive to side effects, thus not allowing for titration to higher doses. The novel finding in our study was that the maladaptive personality traits of Intimacy Avoidance and Deceitfulness predicted less time to discontinuation above and beyond dosage.

More generally, the compelling reasons to expect a role of personality traits in medication adherence and some of the substantial but non-significant effects in this study suggest that future research should examine a wide range of personality traits beyond those that were statistically significant in this study. One important finding from this study is that even when higher order domains are not strong predictors of treatment continuation, lower order facets within those domains may be. This highlights the importance of using relatively comprehensive measures to assess personality in a finer grain than is common in both research and many areas of clinical practice.

Indeed, none of our hypothesized effects were significant. Personality dysfunction was higher in discontinuers, but this effect was not statistically significant either in group differences or survival models. This finding contrasts with results from Haller et al. (2022) who showed that personality dysfunction predicted non-adherence to medication for cardiovascular issues. However, Haller et al. sampled patients with a different clinical problem who were also significantly older compared to our sample, measured adherence and personality functioning differently, and used a different statistical method. Thus, differences across studies may be due to these factors. Gift et al. (2016) found that adults with two more personality disorder diagnoses were more likely to drop out of ADHD treatment. Again, study differences or sampling variability may explain the differences in results. Overall, evidence for the role of personality dysfunction in medication adherence remains mixed and is an important topic for future research.

## Clinical Importance

Possible clinical implications can at least be found in two important areas, the first one concerns the importance of broader personality pathology assessments of the type that can be derived from the Alternative Model of Personality Disorder in the adult ADHD population, and the second is about a closer adherence monitoring of individuals with elevated antagonistic or other interpersonal difficulties. The large discrepancy between the groups concerning stimulant dosage, and whether the recommended max dose was reached, suggests that the discontinued group was less effectively medicated. This could have implications for adherence in itself. Skoglund et al. (2016) found that higher stimulant doses were associated with long-term treatment adherence in individuals with ADHD and Substance Use Disorder.

This emphasizes the importance of assessing personality pathology in individuals with ADHD, as an individualized base to build trust between the patient and the clinicians responsible for administering and monitoring medication so that effective doses are reached. Individuals with higher levels of antagonistic or other mistrust issues, may both: (a) adhere less to medication administration, (b) be more sensitive to side effects, and: (c) elicit more restrictive titration from administering psychiatrists due to the patients being “difficult.” Thus, for clinicians to identify and individualize the contact, building a platform of trust may be even more important for individuals with higher levels of interpersonal dysfunction to optimize pharmacological treatment. In a recent paper, from the large longitudinal Dunedin study sample, Brennan et al. (2024) concluded that mistrust, inferring social distancing, was highly predictive of vulnerability to stressful life events and associated with childhood adversity. Brief psychotherapeutic or alliance-building interventions might be helpful to reinforce pharmacological adherence, as well as targeting specific trust issues. Most psychological treatments include relational aspects in their formats and could thus be integrated with treatment plans for adult ADHD together with pharmacological treatment. An integrative approach thus could theoretically also increase the efficacy of pharmacological treatment of ADHD.

## Limitations and Future Directions

The primary weaknesses of this naturalistic study having to do with sample size, generalizability, and study duration suggest directions for future research.

Our relatively small sample in general and of discontinuers, in particular, may have limited our ability to find statistically significant effects. There was also attrition in some of the analyses due to missing data across cases and compared measures. The attrition rate, however, was balanced and differences were modest. Future research should collect larger samples to achieve adequate statistical power.

In general, rates of discontinuation were somewhat modest compared to previous studies (Brancati et al., 2023; Brikell et al., 2024; Gajria et al., 2014; Schein et al., 2021) and women were somewhat over-represented compared to previous studies of adult ADHD. Furthermore, only 70% of the patients consented to participate, potentially creating selection bias can also be due to patient characteristics such as reading and language ability, decreased stress tolerance, suspiciousness, or patients forgetting to deliver informed consent. It is also not clear how these findings would generalize to other age groups, healthcare systems, or cultures.

Finally, temporary treatment discontinuation may not always indicate a permanent end of treatment. The reinitiation of pharmacological treatment is increasingly common, for example with the increase of diagnosed women in child-bearing ages (Brennan et al., 2024; Brikell et al., 2024). The common heuristic metric to assess discontinuation commonly found in studies is 180 days, which is too short to adequately capture adherence patterns for example in pregnancy and a reasonable post-partum period (Brikell et al., 2024). Thus, the definition of discontinued treatment should in subsequent studies include a reasonably long time frame to account for treatments that may be influenced by adult life choices that result in intentional treatment discontinuation and reinitiation (Rasmussen et al., 2018).

## Conclusion

In this study, we found that adults with ADHD diagnoses were more likely to discontinue medication prematurely related to personality pathology. Our results suggest that adult individuals with ADHD who were intimacy-avoidant and deceitful were more at risk for discontinuation, along with suggestive evidence that other personality variables may be important for predicting treatment continuation as well. The implication that these individuals also did not reach their target dose needs to be explored further, but if true, has important clinical treatment connotations. These findings complement previous research in suggesting the value of personality assessment in patients diagnosed with ADHD and other cognitive or medical disorders. Specific mechanisms underlying these findings are yet to be explored, but our findings indicate that antagonistic features and interpersonal vulnerabilities should be studied further along with interventions directed toward increasing treatment adherence. The broader implication is that the Alternative Model of Personality Disorder has clinical utility for ADHD assessment, treatment monitoring, and how to individualize the relationship between administering clinicians and their patients, and thus make effective treatment more available.

## Supplemental Material

sj-docx-1-jad-10.1177_10870547241309524 – Supplemental material for How Do Personality Dysfunction and Maladaptive Personality Traits Predict Time to Premature Discontinuation of Pharmacological Treatment of ADHD? Supplemental material, sj-docx-1-jad-10.1177_10870547241309524 for How Do Personality Dysfunction and Maladaptive Personality Traits Predict Time to Premature Discontinuation of Pharmacological Treatment of ADHD? by Peter Jacobsson, Tove Granqvist, Christopher J. Hopwood, Robert F. Krueger, Bo Söderpalm and Thomas Nilsson in Journal of Attention Disorders

sj-docx-2-jad-10.1177_10870547241309524 – Supplemental material for How Do Personality Dysfunction and Maladaptive Personality Traits Predict Time to Premature Discontinuation of Pharmacological Treatment of ADHD? Supplemental material, sj-docx-2-jad-10.1177_10870547241309524 for How Do Personality Dysfunction and Maladaptive Personality Traits Predict Time to Premature Discontinuation of Pharmacological Treatment of ADHD? by Peter Jacobsson, Tove Granqvist, Christopher J. Hopwood, Robert F. Krueger, Bo Söderpalm and Thomas Nilsson in Journal of Attention Disorders

sj-docx-3-jad-10.1177_10870547241309524 – Supplemental material for How Do Personality Dysfunction and Maladaptive Personality Traits Predict Time to Premature Discontinuation of Pharmacological Treatment of ADHD? Supplemental material, sj-docx-3-jad-10.1177_10870547241309524 for How Do Personality Dysfunction and Maladaptive Personality Traits Predict Time to Premature Discontinuation of Pharmacological Treatment of ADHD? by Peter Jacobsson, Tove Granqvist, Christopher J. Hopwood, Robert F. Krueger, Bo Söderpalm and Thomas Nilsson in Journal of Attention Disorders

sj-docx-4-jad-10.1177_10870547241309524 – Supplemental material for How Do Personality Dysfunction and Maladaptive Personality Traits Predict Time to Premature Discontinuation of Pharmacological Treatment of ADHD? Supplemental material, sj-docx-4-jad-10.1177_10870547241309524 for How Do Personality Dysfunction and Maladaptive Personality Traits Predict Time to Premature Discontinuation of Pharmacological Treatment of ADHD? by Peter Jacobsson, Tove Granqvist, Christopher J. Hopwood, Robert F. Krueger, Bo Söderpalm and Thomas Nilsson in Journal of Attention Disorders

sj-docx-5-jad-10.1177_10870547241309524 – Supplemental material for How Do Personality Dysfunction and Maladaptive Personality Traits Predict Time to Premature Discontinuation of Pharmacological Treatment of ADHD? Supplemental material, sj-docx-5-jad-10.1177_10870547241309524 for How Do Personality Dysfunction and Maladaptive Personality Traits Predict Time to Premature Discontinuation of Pharmacological Treatment of ADHD? by Peter Jacobsson, Tove Granqvist, Christopher J. Hopwood, Robert F. Krueger, Bo Söderpalm and Thomas Nilsson in Journal of Attention Disorders

sj-docx-6-jad-10.1177_10870547241309524 – Supplemental material for How Do Personality Dysfunction and Maladaptive Personality Traits Predict Time to Premature Discontinuation of Pharmacological Treatment of ADHD? Supplemental material, sj-docx-6-jad-10.1177_10870547241309524 for How Do Personality Dysfunction and Maladaptive Personality Traits Predict Time to Premature Discontinuation of Pharmacological Treatment of ADHD? by Peter Jacobsson, Tove Granqvist, Christopher J. Hopwood, Robert F. Krueger, Bo Söderpalm and Thomas Nilsson in Journal of Attention Disorders
