# Supplementary material for: How Do Personality Dysfunction and Maladaptive Personality Traits Predict Time to Premature Discontinuation of Pharmacological Treatment of ADHD?
Source: J Atten Disord. 2025 Jan 23;29(5):351–62. doi: 10.1177/10870547241309524 (PMC11800730; doi:10.1177/10870547241309524)
Supplement: sj-docx-1-jad-10.1177_10870547241309524 – Supplemental material for How Do Personality Dysfunction and Maladaptive Personality Traits Predict Time to Premature Discontinuation of Pharmacological Treatment of ADHD? [file sj-docx-1-jad-10.1177_10870547241309524.docx]

**Table B. Supplemental material sex and age**

| Distribution of age and sex in samples. Not all individuals included completed the PID-5.  Total N=284; LPFS-BF N=267 (PMD N=54; Continued N=231); PID-5 N=231 (PMD N=49; Continued N=182) | | | | | |
| --- | --- | --- | --- | --- | --- |
|  | p | d | 95% CI | | |
| Age | 0.85 | 0.03 | -0.26 | 0.32 |  |
|  | χ^2^ |  |  |  |  |
| Sex | 0.73 |  |  |  |  |
| *Note:* LPFS-BF = Levels of Personality Functioning Scale, Brief Form; PID-5 = Personality Inventory for the DSM-5; PMD = Prematurely Discontinued | | | | | |
|  |  |  |  |  |  |
|  |  |  |  |  |  |
|  |  |  |  |  |  |
