# Supplementary material for: How Do Personality Dysfunction and Maladaptive Personality Traits Predict Time to Premature Discontinuation of Pharmacological Treatment of ADHD?
Source: J Atten Disord. 2025 Jan 23;29(5):351–62. doi: 10.1177/10870547241309524 (PMC11800730; doi:10.1177/10870547241309524)
Supplement: sj-docx-2-jad-10.1177_10870547241309524 – Supplemental material for How Do Personality Dysfunction and Maladaptive Personality Traits Predict Time to Premature Discontinuation of Pharmacological Treatment of ADHD? [file sj-docx-2-jad-10.1177_10870547241309524.docx]

**Table A. Supplemental material Comorbidity**

| The distribution of co-occurring psychiatric diagnoses in total ADHD sample N = 284 | | |
| --- | --- | --- |
|  | Frequency | Percent |
| Anxiety Disorders | 106 | 37.2 |
| Mood Disorders (Bipolar Disorder) | 78 (14) | 27.4 (4.9) |
| PTSD | 17 | 6.0 |
| Autism Spectrum Disorder | 17 | 6.0 |
| Personality Disorders | 16 | 5.6 |
| Substance Use Disorders | 10 | 3.5 |
| OCD | 9 | 3.2 |
| Eating Disorders | 9 | 3.2 |

*Note:* Diagnoses according to documented ICD-10 codes retrieved from progress notes. PTSD = Post-traumatic Disorder; OCD = Obsessive Compulsive Disorder. Patients can be diagnosed with more than one psychiatric diagnosis, and thereby be included in more than one category.
