# Supplementary material for: How Do Personality Dysfunction and Maladaptive Personality Traits Predict Time to Premature Discontinuation of Pharmacological Treatment of ADHD?
Source: J Atten Disord. 2025 Jan 23;29(5):351–62. doi: 10.1177/10870547241309524 (PMC11800730; doi:10.1177/10870547241309524)
Supplement: sj-docx-3-jad-10.1177_10870547241309524 – Supplemental material for How Do Personality Dysfunction and Maladaptive Personality Traits Predict Time to Premature Discontinuation of Pharmacological Treatment of ADHD? [file sj-docx-3-jad-10.1177_10870547241309524.docx]

**Table C. Supplemental material Internal Consistency**

| Internal consistency in samples. Not all individuals included completed the PID-5.  Total N=284; LPFS-BF N=267 (PMD N=54; Continued N=231); PID-5 N=231 (PMD N=49; Continued N=182) | | | |
| --- | --- | --- | --- |
|  | Total α | PMD α | Continued α |
| **Personality dysfunction (**LPFS-BF) | 0.85 | 0.81 | 0.85 |
| ***Trait Domains and Facets*** (PID-5)  **Negative Affectivity** | 0.91 | 0.91 | 0.91 |
| Emotional Lability | 0.86 | 0.83 | 0.87 |
| Anxiousness | 0.86 | 0.87 | 0.86 |
| Separation Insecurity | 0.86 | 0.87 | 0.86 |
| Restricted Affectivity | 0.79 | 0.84 | 0.76 |
| Hostility | 0.85 | 0.86 | 0.85 |
| Perseveration | 0.81 | 0.82 | 0.81 |
| Submissiveness | 0.78 | 0.76 | 0.79 |
| **Detachment** | 0.92 | 0.93 | 0.91 |
| Withdrawal | 0.92 | 0.92 | 0.91 |
| Anhedonia | 0.82 | 0.82 | 0.82 |
| Intimacy Avoidance | 0.74 | 0.71 | 0.75 |
| Depressivity | 0.94 | 0.96 | 0.93 |
| Suspiciousness | 0.69 | 0.74 | 0.66 |
| **Antagonism** | 0.91 | 0.91 | 0.91 |
| Manipulativeness | 0.81 | 0.77 | 0.83 |
| Deceitfulness | 0.87 | 0.87 | 0.87 |
| Grandiosity | 0.75 | 0.80 | 0.72 |
| Attention Seeking | 0.91 | 0.91 | 0.91 |
| Callousness | 0.88 | 0.90 | 0.87 |
| **Disinhibition** | 0.83 | 0.82 | 0.83 |
| Irresponsibility | 0.56 | 0.57 | 0.55 |
| Impulsivity | 0.78 | 0.70 | 0.80 |
| Distractability | 0.79 | 0.83 | 0.78 |
| Risk Taking | 0.78 | 0.81 | 0.76 |
| Rigid Perfectionism (lack of) | 0.90 | 0.90 | 0.90 |
| **Psychoticism** | 0.94 | 0.95 | 0.94 |
| Unusual Beliefs | 0.82 | 0.83 | 0.81 |
| Eccentricity | 0.94 | 0.93 | 0.94 |
| Perceptual Dysregulation | 0.83 | 0.85 | 0.81 |

*Note.* LPFS-BF = Levels of Personality Functioning Scale, Brief Form; PID-5 = Personality Inventory for the DSM-5; PMD = Prematurely discontinued.
