# Supplementary material for: How Do Personality Dysfunction and Maladaptive Personality Traits Predict Time to Premature Discontinuation of Pharmacological Treatment of ADHD?
Source: J Atten Disord. 2025 Jan 23;29(5):351–62. doi: 10.1177/10870547241309524 (PMC11800730; doi:10.1177/10870547241309524)
Supplement: sj-docx-4-jad-10.1177_10870547241309524 – Supplemental material for How Do Personality Dysfunction and Maladaptive Personality Traits Predict Time to Premature Discontinuation of Pharmacological Treatment of ADHD? [file sj-docx-4-jad-10.1177_10870547241309524.docx]

| **Table D. Supplemental material analyses Covariates** |  |  |  | |
| --- | --- | --- | --- | --- |
|  | p | HR | 95% CI | |
| Max Dose | <.001 | 0.22 | 0.10 | 0.49 |
| AMPH/Day | <.001 | 0.91 | 0.88 | 0.95 |
| Side-effects | 0.10 | 1.61 | 0.92 | 2.82 |
| Comorbidity yes/no | 0.27 | 1.38 | 0.78 | 2.42 |
| Number comorbidity | 0.62 | 1.06 | 0.85 | 1.32 |

*Note:* Total N=269 (prematurely discontinued 52, continued 217)

|  | p | HR | 95% CI | |
| --- | --- | --- | --- | --- |
| Intimacy Avoidance | 0.02 | 1.68 | 1.08 | 2.62 |
| Max Dose | 0.00 | 0.24 | 0.10 | 0.56 |
| Intimacy Avoidance | 0.01 | 1.78 | 1.13 | 2.80 |
| AMPH/Day | <.001 | 0.91 | 0.87 | 0.95 |
| Deceitfulness | 0.03 | 1.64 | 1.04 | 2.60 |
| Max Dose | 0.00 | 0.25 | 0.10 | 0.59 |
| Deceitfulness | 0.04 | 1.63 | 1.03 | 2.57 |
| AMPH/Day | <.001 | 0.91 | 0.88 | 0.95 |

*Note:* Total N=216 (prematurely discontinued 43, continued 173)
