# Supplementary material for: How Do Personality Dysfunction and Maladaptive Personality Traits Predict Time to Premature Discontinuation of Pharmacological Treatment of ADHD?
Source: J Atten Disord. 2025 Jan 23;29(5):351–62. doi: 10.1177/10870547241309524 (PMC11800730; doi:10.1177/10870547241309524)
Supplement: sj-docx-5-jad-10.1177_10870547241309524 – Supplemental material for How Do Personality Dysfunction and Maladaptive Personality Traits Predict Time to Premature Discontinuation of Pharmacological Treatment of ADHD? [file sj-docx-5-jad-10.1177_10870547241309524.docx]

| **Table E. Supplemental Material Descriptive analyses different data sets**  All subjects have not completed all measures. Thus, below are presented analyses in the different data sets so that comparisons can be made across the different data sets**.** | | | | | | | | | | | | | | | | |
| --- | --- | --- | --- | --- | --- | --- | --- | --- | --- | --- | --- | --- | --- | --- | --- | --- |
| **Descriptive statistics Total N=284; LPFS-BF N=267 (PMD N=54; Censored N=231); PID-5 N=231 (PMD N=49; Censored N=182)** | | | | |  | **Descriptive statistics Complete Data Sets N=216**  **(PMD N=46; Censored N=170)** | | | | |  | **Descriptive statistics Complete Data Sets AMPH N=207**  **(PMD N=42; Censored N=165)** | | | | |
|  | p | d | Lower | Upper |  |  | p | d | Lower | Upper |  |  | p | d | Lower | Upper |
| LPFS-BF | 0.08 | ***0.27*** | -0.03 | 0.57 |  | LPFS-BF | **0.04** | **0.35** | 0.02 | 0.68 |  | LPFS-BF | **0.04** | **0.35** | 0.01 | 0.69 |
| Negative Affectivity | 1.00 | 0.00 | -0.32 | 0.32 |  | Negative Affectivity | 0.94 | -0.01 | -0.34 | 0.31 |  | Negative Affectivity | 0.66 | -0.08 | -0.42 | 0.26 |
| Antagonism | **0.03** | **0.36** | 0.04 | 0.67 |  | Antagonism | **0.03** | **0.37** | 0.05 | 0.70 |  | Antagonism | **0.03** | **0.37** | 0.03 | 0.71 |
| Disinhibition | 0.49 | 0.11 | -0.21 | 0.43 |  | Disinhibition | 0.42 | 0.14 | -0.19 | 0.46 |  | Disinhibition | 0.39 | 0.15 | -0.19 | 0.49 |
| Detachment | 0.35 | 0.15 | -0.16 | 0.47 |  | Detachment | 0.14 | 0.25 | -0.08 | 0.57 |  | Detachment | 0.28 | 0.19 | -0.15 | 0.53 |
| Psychoticism | 0.27 | 0.18 | -0.14 | 0.49 |  | Psychoticism | 0.17 | 0.23 | -0.10 | 0.56 |  | Psychoticism | 0.31 | 0.18 | -0.16 | 0.52 |
| Submissiveness | 0.34 | -0.15 | -0.47 | 0.16 |  | Submissiveness | 0.22 | -0.21 | -0.53 | 0.12 |  | Submissiveness | 0.36 | -0.16 | -0.50 | 0.18 |
| Depressivity | 0.67 | 0.07 | -0.25 | 0.38 |  | Depressivity | 0.73 | 0.06 | -0.27 | 0.38 |  | Depressivity | 0.90 | 0.02 | -0.32 | 0.36 |
| Separation Insecurity | 0.62 | -0.08 | -0.39 | 0.24 |  | Separation Insecurity | 0.56 | -0.10 | -0.42 | 0.23 |  | Separation Insecurity | 0.48 | -0.12 | -0.46 | 0.22 |
| Anxiousness | 0.92 | 0.02 | -0.30 | 0.33 |  | Anxiousness | 0.97 | 0.01 | -0.32 | 0.33 |  | Anxiousness | 0.75 | -0.06 | -0.39 | 0.28 |
| Emotional Lability | 0.66 | 0.07 | -0.25 | 0.39 |  | Emotional Lability | 0.72 | 0.06 | -0.27 | 0.39 |  | Emotional Lability | 0.98 | -0.01 | -0.34 | 0.33 |
| Suspiciousness | 0.60 | 0.08 | -0.23 | 0.40 |  | Suspiciousness | 0.88 | 0.03 | -0.30 | 0.35 |  | Suspiciousness | 0.76 | 0.05 | -0.29 | 0.39 |
| Restricted Affectivity | 0.84 | 0.03 | -0.28 | 0.35 |  | Restricted Affectivity | 0.51 | 0.11 | -0.22 | 0.44 |  | Restricted Affectivity | 0.67 | 0.08 | -0.26 | 0.41 |
| Withdrawal | 0.42 | 0.13 | -0.19 | 0.45 |  | Withdrawal | 0.25 | 0.19 | -0.14 | 0.52 |  | Withdrawal | 0.50 | 0.12 | -0.22 | 0.46 |
| Intimacy Avoidance | **0.04** | **0.34** | 0.02 | 0.66 |  | Intimacy Avoidance | **0.01** | **0.42** | 0.10 | 0.75 |  | Intimacy Avoidance | **0.02** | **0.40** | 0.06 | 0.74 |
| Anhedonia | 0.53 | -0.10 | -0.42 | 0.22 |  | Anhedonia | 0.87 | -0.03 | -0.35 | 0.30 |  | Anhedonia | 0.74 | -0.06 | -0.40 | 0.28 |
| Manipulativeness | 0.14 | ***0.24*** | -0.08 | 0.56 |  | Manipulativeness | 0.11 | ***0.27*** | -0.06 | 0.59 |  | Manipulativeness | 0.15 | ***0.25*** | -0.09 | 0.59 |
| Deceitfulness | **0.01** | **0.41** | 0.09 | 0.73 |  | Deceitfulness | **0.02** | **0.40** | 0.07 | 0.72 |  | Deceitfulness | **0.02** | **0.42** | 0.08 | 0.76 |
| Hostility | **0.02** | **0.39** | 0.07 | 0.71 |  | Hostility | **0.02** | **0.39** | 0.06 | 0.72 |  | Hostility | **0.04** | **0.35** | 0.01 | 0.69 |
| Callousness | 0.10 | ***0.27*** | -0.05 | 0.58 |  | Callousness | 0.07 | ***0.31*** | -0.02 | 0.63 |  | Callousness | 0.07 | ***0.31*** | -0.03 | 0.65 |
| Attention Seeking | 0.59 | 0.09 | -0.23 | 0.40 |  | Attention Seeking | 0.60 | 0.09 | -0.24 | 0.41 |  | Attention Seeking | 0.64 | 0.08 | -0.26 | 0.42 |
| Grandiosity | 0.10 | ***0.27*** | -0.05 | 0.58 |  | Grandiosity | 0.06 | ***0.31*** | -0.02 | 0.64 |  | Grandiosity | 0.07 | ***0.31*** | -0.03 | 0.65 |
| Irresponsibility | 0.19 | ***0.21*** | -0.11 | 0.53 |  | Irresponsibility | 0.16 | ***0.23*** | -0.10 | 0.56 |  | Irresponsibility | 0.17 | ***0.24*** | -0.10 | 0.58 |
| Impulsivity | 0.23 | 0.19 | -0.12 | 0.51 |  | Impulsivity | 0.16 | ***0.23*** | -0.09 | 0.56 |  | Impulsivity | 0.16 | ***0.24*** | -0.10 | 0.58 |
| Distractability | 0.29 | -0.17 | -0.49 | 0.14 |  | Distractability | 0.28 | -0.18 | -0.51 | 0.15 |  | Distractability | 0.34 | -0.16 | -0.50 | 0.18 |
| Perseveration | 0.66 | -0.07 | -0.39 | 0.24 |  | Perseveration | 0.47 | -0.12 | -0.45 | 0.21 |  | Perseveration | 0.32 | -0.17 | -0.51 | 0.17 |
| Rigid Perfectionism | 0.84 | 0.03 | -0.28 | 0.35 |  | Rigid Perfectionism | 0.98 | 0.01 | -0.32 | 0.33 |  | Rigid Perfectionism | 0.55 | -0.10 | -0.44 | 0.24 |
| Risk Taking | 0.66 | 0.07 | -0.24 | 0.39 |  | Risk Taking | 0.50 | 0.11 | -0.21 | 0.44 |  | Risk Taking | 0.45 | 0.13 | -0.21 | 0.47 |
| Eccentricity | 0.51 | 0.11 | -0.21 | 0.42 |  | Eccentricity | 0.33 | 0.16 | -0.16 | 0.49 |  | Eccentricity | 0.58 | 0.10 | -0.24 | 0.44 |
| Perceptual Dysregulation | 0.15 | ***0.23*** | -0.08 | 0.55 |  | Perceptual Dysregulation | 0.10 | ***0.28*** | -0.05 | 0.61 |  | Perceptual Dysregulation | 0.12 | ***0.27*** | -0.07 | 0.61 |
| Unusual Beliefs | 0.31 | 0.17 | -0.15 | 0.48 |  | Unusual Beliefs | 0.28 | 0.18 | -0.15 | 0.51 |  | Unusual Beliefs | 0.48 | 0.12 | -0.22 | 0.46 |
|  |  |  |  |  |  |  |  |  |  |  |  |  |  |  |  |  |

***Note.*** LPFS-BF = Levels of Personality Functioning Scale, Brief Form; PID-5 = Personality Inventory for the DSM-5**; Bold** signify correlations significantly different from zero (p < 0.05). **Bold** and *italicized* signify effect sizes above d=0.2. *Italicized* facets are the primary facets included in the APA algorithms för higher order domains.
