# Supplementary material for: How Do Personality Dysfunction and Maladaptive Personality Traits Predict Time to Premature Discontinuation of Pharmacological Treatment of ADHD?
Source: J Atten Disord. 2025 Jan 23;29(5):351–62. doi: 10.1177/10870547241309524 (PMC11800730; doi:10.1177/10870547241309524)
Supplement: sj-docx-6-jad-10.1177_10870547241309524 – Supplemental material for How Do Personality Dysfunction and Maladaptive Personality Traits Predict Time to Premature Discontinuation of Pharmacological Treatment of ADHD? [file sj-docx-6-jad-10.1177_10870547241309524.docx]

**Table F. Supplemental material Hazard Ratios Different Data Sets**

| All subjects have not completed all measures. Thus, below are presented analyses in the different data sets so that comparisons can be made across the different data sets | | | | | | | | | | | | | | | | |
| --- | --- | --- | --- | --- | --- | --- | --- | --- | --- | --- | --- | --- | --- | --- | --- | --- |
| **Cox HR Univariate Total N=284 Total N=284;  LPFS-BF N=267 (PMD N=54; Censored N=231); PID-5 N=231 (PMD N=49; Censored N=182)** | | | | |  | **Cox HR Univariate Complete Data Sets N=216 (PMD N=46; Censored N=170)** | | | | |  | **Cox HR Univariate Complete Data Sets AMPH N=207 (PMD N=42; Censored N=165)** | | | | |
|  | p | HR | CI 95% | |  |  | p | HR | CI 95% | |  |  | p | HR | CI 95% | |
| LPFS-BF | 0.09 | ***1.44*** | 0.94 | 2.20 |  | LPFS-BF | 0.06 | ***1.57*** | 0.98 | 2.52 |  | LPFS-BF | 0.06 | ***1.61*** | 0.98 | 2.65 |
| Negative Affectivity | 0.90 | 0.97 | 0.61 | 1.53 |  | Negative Affectivity | 0.87 | 0.96 | 0.60 | 1.55 |  | Negative Affectivity | 0.65 | 0.89 | 0.54 | 1.48 |
| Detachment | 0.18 | ***1.46*** | 0.85 | 2.52 |  | Detachment | 0.06 | ***1.69*** | 0.97 | 2.93 |  | Detachment | 0.14 | ***1.56*** | 0.87 | 2.78 |
| Antagonism | 0.06 | ***1.57*** | 0.98 | 2.50 |  | Antagonism | 0.07 | ***1.56*** | 0.96 | 2.54 |  | Antagonism | 0.07 | ***1.59*** | 0.96 | 2.65 |
| Disinhibition | 0.84 | 1.07 | 0.54 | 2.11 |  | Disinhibition | 0.77 | 1.11 | 0.55 | 2.25 |  | Disinhibition | 0.72 | 1.14 | 0.55 | 2.37 |
| Psychoticism | 0.55 | 1.17 | 0.70 | 1.95 |  | Psychoticism | 0.35 | 1.28 | 0.76 | 2.17 |  | Psychoticism | 0.56 | 1.18 | 0.68 | 2.06 |
| Submissiveness | 0.67 | 0.92 | 0.61 | 1.37 |  | Submissiveness | 0.48 | 0.86 | 0.58 | 1.29 |  | Submissiveness | 0.71 | 0.92 | 0.60 | 1.41 |
| Depressivity | 0.58 | 1.11 | 0.77 | 1.60 |  | Depressivity | 0.66 | 1.09 | 0.75 | 1.59 |  | Depressivity | 0.79 | 1.06 | 0.71 | 1.57 |
| Separation Insecurity | 0.59 | 0.90 | 0.63 | 1.30 |  | Separation Insecurity | 0.60 | 0.90 | 0.61 | 1.33 |  | Separation Insecurity | 0.54 | 0.88 | 0.58 | 1.33 |
| Anxiousness | 0.87 | 0.97 | 0.66 | 1.41 |  | Anxiousness | 0.79 | 0.95 | 0.64 | 1.40 |  | Anxiousness | 0.59 | 0.89 | 0.59 | 1.35 |
| Emotional Lability | 0.64 | 1.09 | 0.75 | 1.59 |  | Emotional Lability | 0.70 | 1.08 | 0.73 | 1.59 |  | Emotional Lability | 0.96 | 1.01 | 0.67 | 1.52 |
| Suspiciousness | 0.61 | 1.14 | 0.70 | 1.86 |  | Suspiciousness | 0.87 | 1.04 | 0.62 | 1.75 |  | Suspiciousness | 0.77 | 1.08 | 0.63 | 1.86 |
| Restricted Affectivity | 0.91 | 0.97 | 0.63 | 1.51 |  | Restricted Affectivity | 0.74 | 1.08 | 0.69 | 1.70 |  | Restricted Affectivity | 0.88 | 1.04 | 0.64 | 1.67 |
| Withdrawal | 0.38 | 1.19 | 0.81 | 1.74 |  | Withdrawal | 0.22 | 1.28 | 0.86 | 1.91 |  | Withdrawal | 0.42 | 1.19 | 0.78 | 1.82 |
| Intimacy Avoidance | **0.00** | **1.88** | 1.23 | 2.89 |  | Intimacy Avoidance | **<.001** | **2.07** | 1.34 | 3.18 |  | Intimacy Avoidance | ***0.00*** | ***2.02*** | 1.29 | 3.17 |
| Anhedonia | 0.67 | 0.91 | 0.58 | 1.42 |  | Anhedonia | 0.93 | 0.98 | 0.62 | 1.55 |  | Anhedonia | 0.81 | 0.94 | 0.58 | 1.52 |
| Manipulativeness | 0.28 | 1.23 | 0.85 | 1.78 |  | Manipulativeness | 0.30 | 1.22 | 0.84 | 1.79 |  | Manipulativeness | 0.34 | 1.21 | 0.81 | 1.81 |
| Deceitfulness | **0.03** | **1.61** | 1.05 | 2.45 |  | Deceitfulness | 0.07 | ***1.51*** | 0.97 | 2.34 |  | Deceitfulness | 0.05 | ***1.59*** | 1.01 | 2.53 |
| Hostility | 0.08 | ***1.47*** | 0.95 | 2.28 |  | Hostility | 0.10 | ***1.46*** | 0.93 | 2.27 |  | Hostility | 0.15 | ***1.41*** | 0.88 | 2.24 |
| Callousness | 0.18 | ***1.41*** | 0.86 | 2.33 |  | Callousness | 0.14 | ***1.47*** | 0.88 | 2.45 |  | Callousness | 0.13 | ***1.50*** | 0.89 | 2.56 |
| Attention Seeking | 0.81 | 1.04 | 0.74 | 1.47 |  | Attention Seeking | 0.83 | 1.04 | 0.73 | 1.49 |  | Attention Seeking | 0.84 | 1.04 | 0.72 | 1.51 |
| Grandiosity | 0.10 | ***1.47*** | 0.93 | 2.32 |  | Grandiosity | 0.05 | ***1.61*** | 1.00 | 2.60 |  | Grandiosity | 0.06 | ***1.62*** | 0.99 | 2.65 |
| Irresponsibility | 0.37 | 1.27 | 0.75 | 2.16 |  | Irresponsibility | 0.33 | 1.32 | 0.76 | 2.28 |  | Irresponsibility | 0.33 | 1.33 | 0.75 | 2.35 |
| Impulsivity | 0.51 | 1.17 | 0.74 | 1.85 |  | Impulsivity | 0.45 | 1.20 | 0.75 | 1.93 |  | Impulsivity | 0.44 | 1.22 | 0.74 | 2.01 |
| Distractability | 0.21 | 0.71 | 0.42 | 1.21 |  | Distractability | 0.22 | 0.71 | 0.41 | 1.23 |  | Distractability | 0.28 | 0.73 | 0.41 | 1.30 |
| Perseveration | 0.40 | 0.82 | 0.52 | 1.30 |  | Perseveration | 0.34 | 0.80 | 0.50 | 1.28 |  | Perseveration | 0.24 | 0.74 | 0.45 | 1.22 |
| Rigid Perfectionism | 0.68 | 0.93 | 0.64 | 1.34 |  | Rigid Perfectionism | 0.63 | 0.91 | 0.62 | 1.33 |  | Rigid Perfectionism | 0.30 | 0.81 | 0.54 | 1.21 |
| Risk Taking | 0.89 | 0.97 | 0.57 | 1.63 |  | Risk Taking | 1.00 | 1.00 | 0.59 | 1.69 |  | Risk Taking | 0.94 | 1.02 | 0.59 | 1.76 |
| Eccentricity | 0.99 | 1.00 | 0.70 | 1.43 |  | Eccentricity | 0.67 | 1.08 | 0.75 | 1.56 |  | Eccentricity | 0.96 | 1.01 | 0.69 | 1.48 |
| Perceptual Dysregulation | 0.27 | 1.33 | 0.80 | 2.22 |  | Perceptual Dysregulation | 0.16 | ***1.44*** | 0.87 | 2.40 |  | Perceptual Dysregulation | 0.20 | 1.42 | 0.83 | 2.42 |
| Unusual Beliefs | 0.42 | 1.21 | 0.76 | 1.95 |  | Unusual Beliefs | 0.42 | 1.23 | 0.75 | 2.02 |  | Unusual Beliefs | 0.67 | 1.13 | 0.66 | 1.94 |
|  |  |  |  |  |  |  |  |  |  |  |  |  |  |  |  |  |

*Note.* LPFS-BF = Levels of Personality Functioning Scale, Brief Form; PID-5 = Personality Inventory for the DSM-5; **Bold** signify hazard ratios significantly different from zero (p < 0.05). **Bold** and *italized* signify hazard ratios over 40%. *Italicized* facets are the primary facets included in the APA algorithms för higher order domains.
